# Urinary Microbiome Profiling by Shotgun Metagenomic Sequencing in Women Having Acute Cystitis-Like Symptoms With Negative Urine Cultures

**DOI:** 10.7759/cureus.100451

**Published:** 2025-12-30

**Authors:** Masanori Matsukawa, Yasuyuki Sakai, Kotaro Aoki, Yoshikazu Ishii

**Affiliations:** 1 Department of Urology, Takikawa Municipal Hospital, Takikawa, JPN; 2 Department of Urology, Sapporo Medical University, Sapporo, JPN; 3 Department of Microbiology and Infectious Diseases, Toho University School of Medicine, Tokyo, JPN; 4 Department of Microbial Genomics and Ecology, Center for the Planetary Health and Innovation Science, The IDEC Institute, Hiroshima University, Higashihiroshima, JPN

**Keywords:** acute cystitis, culture negative, lower urinary tract infections, microbiome, shotgun metagenomic sequencing

## Abstract

Background: Women presenting with typical symptoms of acute cystitis but with negative urine cultures, termed acute cystitis-like symptoms with negative urine cultures (ACNCs) in this study, are not uncommon. Despite previous attempts to detect bacterial DNA in urine, the etiology remains unclear. Although alterations in the urinary microbiome have been linked to other urological disorders, its involvement in ACNC has not been thoroughly investigated.

Methods: Between September 2016 and December 2017, midstream urine samples were collected from women aged ≥16 years who had at least one typical symptom of acute cystitis and a negative urine culture. Samples were obtained at the initial (V1) and follow-up (V2) visits. Shotgun metagenomic sequencing (SMG) was performed via an Illumina MiSeq system. Taxonomic analysis at the genus level included taxa with ≥10 assigned reads in samples with ≥10,000 human-subtracted reads (HSRs).

Results: Of 206 eligible women, 15 (7.3%; median age, 65 years) met the ACNC criteria and were enrolled. SMG was conducted for 15 samples at V1 and nine samples at V2. At V1, the HSR varied widely, and only five samples met the criteria for reliable interpretation. Seven samples, particularly those with high-grade pyuria, contained fewer than 1,000 HSRs, indicating potentially very low microbial loads or technical limitations. ACNC microbiomes demonstrated marked interindividual variation in taxonomic composition. The predominant taxa most frequently observed were *Lactobacillus *spp., *Gardnerella *spp., and JC polyomavirus. Conventional uropathogens, such as *Escherichia *spp.*,* were not identified at interpretable levels. At V2, microbial diversity remained heterogeneous, but eight samples yielded sufficient read counts for interpretation.

Conclusions: While conventional uropathogens below interpretable criteria are unlikely to be responsible for most ACNCs, it is not necessarily recommended to regard the leading taxon in each case as the cause or to exclude microbiological involvement simply due to a low HSR because no validated metagenomic signature distinguishes pathogens from commensals. However, the observed diversity in ACNC microbiome profiles may reflect a heterogenous group of microbial conditions, including potentially viral, and nonmicrobial etiologies.

## Introduction

Acute cystitis is one of the most common bacterial infections in women [1]. Its diagnosis is typically based on characteristic symptoms, such as dysuria and increased urinary frequency, in combination with pyuria [2]. Although a positive urine culture for significant growth of bacteria remains the gold standard for identifying causative pathogens [3], it is not uncommon for patients presenting with typical symptoms of acute cystitis to have negative urine cultures [4,5]. This condition has been referred to as abacterial cystitis [6], urethral syndrome [7], or women with symptoms of a urinary tract infection but a negative urine culture [8]. However, these terms may not be entirely appropriate, as they imply a lack of bacterial involvement despite the possibility of unculturable status or suggest urethral localization despite symptoms that may not be confined to the urethra. Furthermore, the term "urinary tract infection" encompasses infections of both the lower and upper urinary tract. Therefore, in this study, we adopted the term acute cystitis-like symptoms with negative urine cultures (ACNCs) to more accurately describe this condition.

There have been controversies about the etiology of ACNCs. Important advances in this field include a study by Heytens et al. [8], which demonstrated that *Escherichia coli *DNA can be detected in the majority of ACNC cases via quantitative polymerase chain reaction (qPCR) targeting the *E. coli* b-glucuronidase encoding gene *uidA*, *Staphyolococcus saprophyticus* *trk* region, *Chlamydia trachomatis*, *Neisseria gonorrhoeae*, *Mycoplasma genitalium*, and *Trichomonas vaginalis* in urine samples. However, although *E. coli* is the major uropathogenic bacterium, limited microorganisms have been targeted for detection, leaving the potential involvement of other bacteria unclear. Moreover, normal urine is not sterile [9,10], and the human urinary tract harbors a unique microbiome with high interindividual variability [11]. This indicates that the mere detection of bacterial DNA in urine does not necessarily identify the causative agent of ACNCs. Taken together, these observations suggest that the true etiology of ACNCs remains uncertain, necessitating further investigation via unbiased approaches, such as metagenomic sequencing. Despite growing evidence of the role of the urinary microbiome in various urinary tract conditions [9,12], the composition and dynamics of the microbiome in ACNCs remain poorly understood. The objective of this study was to characterize the urinary microbiome of women with ACNCs using shotgun metagenomic sequencing (SMG) and to explore potential microbial signatures associated with this condition.

## Materials and methods

Patients

The patients included women with symptoms of acute cystitis who visited the outpatient clinics of Takikawa Municipal Hospital from September 2016 to December 2017 and provided written informed consent for inclusion in this study. The inclusion and exclusion criteria are listed in Table 1. All the patients received antimicrobial therapy at the initial visit (V1) in accordance with current clinical guidelines and practice. Age, history of acute cystitis, a profile of the chief complaints, antibacterial agents used, and treatment response were recorded. The patients were instructed to return to the clinic in two weeks. The response was judged unfavorable if the patient did not feel cured or required additional treatment; otherwise, it was considered favorable.

**Table 1:** The inclusion and exclusion criteria. WBCs: white blood cells; HPF: high-power field.

Criteria
Inclusion
Female sex
Age ≥16 years
Presence of at least one of the following symptoms as the chief complaint
Painful or uncomfortable urination
Suprapubic pain or discomfort
Increased urinary frequency
Feeling of incomplete bladder emptying
Symptom duration <4 weeks
Pyuria ≥10 WBCs/HPF
Negative urine culture
Exclusion
Antimicrobial agent use within the previous 4 weeks
Vaginal discharge
Vaginal irritative symptoms
Signs of pyelonephritis (fever or flank pain)
History of underlying urological diseases

Urine samples collection, testing, and storage

Collection

Patients were instructed to spread the labia, cleanse their external genitalia with a cotton sheet soaked in 5% benzalkonium chloride, and then collect a midstream urine sample.

Testing

At V1, the urine was subjected to urinalysis and bacterial culture (described below). At the follow-up visit (V2), urinalysis was performed.

Storage

Ten milliliters of urine collected from patients at V1 and V2 were placed into sterile polypropylene test tubes (ASIAKIZAI Inc., Japan), frozen at -20°C within two hours of collection at Takikawa Municipal Hospital, and sent to Toho University for subsequent DNA extraction. SMG was performed on samples from both visits when the culture at V1 was negative.

Bacterial isolation and identification

Urine Culture

Urine culture was performed by inoculating 10 µl of urine onto a 5% sheep blood agar plate (Nissui Pharmaceutical Co., Japan) and a modified Drigalski lactose agar plate (Kyokuto Pharmaceutical Industrial Co., Japan). The urine samples were also applied to Diaslide urine culture devices (Nissui Pharmaceutical Co., Japan) [13]. The plates and devices were incubated aerobically at 37°C for 18 hours. If the results of the aerobic culture and Gram stain were discrepant, additional anaerobic incubation in 80% N_2_, 10% H_2_, and 10% CO_2_ in the presence of sulfate-copper saturated steel wool for oxygen absorption was also performed at 37˚C for 48 hours [14]. Viable bacteria were enumerated with the Diaslide device, and more than one colony-forming unit per ml (equivalent to 1,000 colony-forming units/ml) for bacteria was considered positive. The bacterial species were identified with the Microscan WalkAway-96 System (Beckman Coulter, USA) according to the manufacturer's instructions.

Shotgun metagenomic sequencing

DNA Extraction

DNA was extracted from up to 10 ml of each urine sample. The urine sample was centrifuged at 2,500×g for 10 minutes and then the supernatant was discarded, leaving 200 µl of bacteria-containing precipitate. To increase DNA recovery, the bacteria-containing precipitate was physically crushed for one minute at maximum speed in a tabletop vortex mixer using Easy-Beads (AMR, Japan). The DNA was eluted in 50 µl of ultrapure water via a DNA mini kit (QIAGEN, Germany) according to the instruction manual.

DNA Library Preparation and Sequencing Protocol

DNA library preparation for MiSeq (Illumina, USA) was performed via Nextera XT (Illumina, USA). Single-end 150-mer reads were obtained via a MiSeq Reagent Kit v3 150 cycle kit (Illumina, USA). Adapter and quality trimming was performed via fastp [15], which involves trimming five bases at the 5’ end and filtering out reads of less than the quality scores of 20 (Q20) and 50-mer. The human genome was removed via KneaData (https://github.com/biobakery/kneaddata) with the hg37, which assembled the human genome as the reference sequence. The origin of each read was analyzed via BBtools (https://github.com/BioInfoTools/BBMap), which compares the Ketch command with reference to the National Center for Biotechnology Information (NCBI) Refseq database downloaded on May 21, 2022. MegaHit [16] was used for the de novo assembly of the HSR. Similarity searches of the assembled genome with known sequences were performed via NCBI nucleotide BLAST (https://blast.ncbi.nlm.nih.gov/Blast.cgi). 

Ethics

The study was conducted according to the Declaration of Helsinki, national and institutional standards, and STROME-ID guidelines [17]. Ethical approval for this study was granted by the Ethics Committees of Takikawa Municipal Hospital (No. 15-10) and of the Faculty of Medicine, Toho University (A16053 and A19041).

## Results

Patients

A total of 206 women presented to our clinic with symptoms that met our inclusion criteria. Of these, 15 (7.3%) had negative urine cultures and were included in the study. The clinical characteristics are summarized in Table 2. The median age of the study participants was 65 years, with the majority reporting a past history of acute cystitis. The most common symptom was painful or uncomfortable urination. All patients received antimicrobial therapy, with 13 of the 15 patients demonstrating a favorable clinical response.

**Table 2:** Clinical characteristics and microscopic urinary findings of the study subjects. Estimates were given as median (quartile 1, quartile 3) or frequency (percentage). UTI: urinary tract infections; WBC: white blood cell; RBC: red blood cell; SEC: squamous epithelial cell; hpf: high power field. ^*^Levofloxacin, fosfomycin, and trimethoprim-sulfamethoxazole. ^†^Number of white blood cells, red blood cells, and epithelial cells were determined by automated urine particle analyzer (UF-5000; Sysmex Corporation, Kobe, Japan) and confirmed by unstained microscopic examination.

Variables	(n = 15)
Age	65 (51, 68)
Past history of UTI, n (%)	12 (80)
Symptoms at the first visit, n (%)	
Painful or uncomfortable urination	12 (80)
Frequency	8 (53)
Suprapubic pain or discomfort	1 (6.7)
Incomplete emptying feeling	2 (13)
Antimicrobials, n (%)	
Cephalosporins	6 (40)
Penicillin	3 (20)
Others*	6 (40)
Response to treatment, n (%)	
Favorable	13 (87)
Unfavorable	2 (13)
Urinary sediments	
WBCs/hpf, n (%)^†^	
5-9	5 (33)
10-49	5 (33)
≥50	5 (33)
RBCs/hpf, n (%)^†^	
<5	6 (40)
≥5	9 (60)
SECs/hpf, n (%)^†^	
<1	5 (33)
≥1	10 (67)

Shotgun metagenomic sequencing

Sample Processing and Yield of Read

Urine samples were collected at Takikawa Municipal Hospital and initially stored at -20 °C for one to seven months. They were then transferred to Toho University, where they were stored at -80 °C for an additional 3 to 3.5 years prior to DNA extraction. Shotgun metagenomic sequencing (SMG) was performed on 15 samples at V1 and nine at V2 (Table 5 in Appendices). The number of human-subtracted reads (HSRs) varied widely across the cases (Table 5 in Appendices). At V1, seven cases (A, B, E, G, I, K, and M) had fewer than 1,000 HSRs, whereas three (F, J, and O) had between 1,000 and 10,000 HSRs. Only five cases (C, D, H, L, and N) exceeded 10,000 HSRs, a threshold [18,19] defined post hoc for reliable taxonomic evaluation (Table 3 and Table 6 in Appendices). Notably, four of the five cases (Cases A, E, G, and I) with high-grade pyuria (≥50 WBCs/HPF) had fewer than 1,000 HSRs, with Case N being the exception.

**Table 3:** Identified genera at the initial visit by SMG. The numbers in parentheses indicate either the sum of assigned reads per case or the assigned reads per taxa. S: number of genera with interpretable level of assigned read count; SMG: shotgun metagenomic sequencing. ^*^Propionimicrobium spp. (9), *Finegoldia* spp. (9), *Varibaculum* spp. (8), and *Bacteroides* spp. (7). ^†^*Aerococcus* spp. (9), *Chlamydia* spp. (6), and *Sphingomonas* spp. (5). ^‡^*Bacteroides* spp. (8), *Facklamia* spp. (8), *Varibaculum* spp. (8),* Anaerosphaera* spp. (6), *Prevotella* spp. (5), *Dialiste*r spp. (3), *Actinotignum* spp. (3), and *Urinacoccu*s spp. (3).

Case	Relative abundance				S
	≥50%	≥10% to <50%	≥5% to <10%	≥1% to <5%	
C (616)	JC polyomavirus (318)	*Prevotella* spp. (95)	*Porphyromonas* spp. (38)	*Actinomyces* spp. (27), *Metaprevotella* spp. (23), *Anaerosphaera* spp. (20), *Peptoniphilus* spp. (14), *Anaerococcus* spp. (14), 4 taxa* (< 10 reads)	8
D (10469)	*Gardnerella* spp.(6978)	*Atopobium* spp. (3252)			2
H (1031)	*Lactobacillus* spp. (999)			*Chlamydia* spp. (12),* Neisseria* spp. (10)	3
L (301)	*Lactobacillus* spp. (225)	*Gardnerella* spp. (35)	JC polyomavirus (21)	3 taxa^†^ (<10 reads)	3
N (269)		*Porphyromonas* spp. (105), *Peptoniphilus* spp. (30)	*Propionimicrobium* spp. (26), *Actinomyces* spp. (19), *Ezakiella* spp. (16)	*Anaerococcus* spp. (10), 8 taxa^‡^ (<10 reads)	6

At V2, SMG was conducted in nine cases (C, E, F, G, H, I, J, L, and O), none of which had pyuria ≥10 WBCs/HPF (Table 7 in Appendices). Eight cases (all except L) had more than 10,000 HSRs (Table 4). However, only two cases (C and H) had more than 10,000 HSRs at both V1 and V2, limiting the number of cases available for direct taxonomic comparison before and after treatment in this study.

In addition to the threshold of ≥10,000 HSRs in a sample for taxonomic evaluation, only taxa with a minimum assigned reads of 10 were included [18,19] in further analysis as interpretable taxa. The number of assigned reads was used as a surrogate for relative abundance, not absolute quantification. The bacterial taxonomy was assessed at the genus level.

Taxonomic Profiles at V1

Genus composition and richness varied significantly among cases at both V1 and V2 (Tables 3, 4). At V1, the taxon diversity varied considerably (Table 3). In Case C, JC polyomavirus (JCV) was predominant in 52% of the eight taxa, followed by *Prevotella* spp. and *Porphyromonas* spp. In Case D, *Gardnerella* was the most abundant (67%) among the two taxa, followed by *Atopobium* spp. In both Cases H and L, *Lactobacillus* spp. was the predominant taxon, comprising 97% and 70% of the total reads, respectively. Each case contained three taxa. In Case H, the remaining taxa, *Chlamydia* spp. and *Neisseria* spp. were detected at low relative abundance. In Case L, *Gardnerella* spp. and JCV were the second and third most abundant taxa. In Case N, six taxa exhibited a balanced distribution, with *Porphyromonas* spp. being the most abundant, constituting less than half of the total reads. In five cases at V1, *Escherichia* spp. and other conventional uropathogenic bacteria were not identified at the interpretable level. On the other hand, in four of the five cases, three taxa, *Lactobacillus* spp., *Gardnerella* spp., and JCV, were isolated with the highest abundance (Table 3 and Tables 5, 6 in Appendices).

**Table 4:** Identified genera at the follow-up visit by SMG. The numbers in parentheses indicate either the total assigned reads per case or per genus. S: number of genera with interpretable level of assigned read count; S’: number of genera less than 1% of relative abundance but with interpretable level of assigned read count; SMG: shotgun metagenomic sequencing. ^*^Propionimicrobium spp. (9). ^†^*Citrobacter* spp. (9), *Clostridium* spp. (9), *Peptoniphilus* spp. (9), *Salmonella* spp. (7). ^‡^*Metaprevotella* spp. (7). ^§^*Bacteroides* spp. (40), *Lawsonella* spp. (29), *Streptococcus* spp. (27), *Actinotignum *spp. (17), *Alistipes* spp. (17), *Varibaculum* spp. (17), *Collinsella* spp. (16), JC polyomavirus (13), *Actinomyces* spp. (11), *Alloscardovia* spp. (11), *Ruminococcus* spp. (10). ^||^*Staphylococcus* spp. (10). ^¶^*Streptobacillus* spp. (17), *Anaerococcus* spp. (14), *Veillonella* spp. (12), *Fenollaria* spp. (11).

Case	Relative abundance				S, S’
	≥50%	≥10% to <50%	≥5% to <10%	≥1% to <5%	
C (831)	JC polyomavirus (456)	*Prevotella* spp. (238)	*Metaprevotella* spp. (71), *Porphyromona* spp. (42)	1 taxon* (<10 reads)	4, 0
E (405)		*Ruminococcus* spp. (64),* Staphylococcus* spp. (48), *Escherichia* spp. (47), *Enterococcus* spp. (42), *Bacteroides* spp. (41)	*Finegoldia* spp. (24)	*Streptococcus* spp. (19), *Alistipes* spp. (10), 4 taxa^†^ (<10 reads)	7, 0
F (4727)	JC polyomavirus (4717)				1, 0
G (6727)		*Prevotella* spp. (2869), Porphyromonas spp. (2446)	*Propionimicrobium* spp. (671), *Metaprevotella* spp. (508)		15, 11^§^
H (1061)	*Lactobacillus* spp. (993)			*Ureaplasma* spp. (30), *Chlamydia* spp. (11)	4, 1^||^
I (612)	*Streptococcus* spp. (318)	*Prevotella* spp. (126), *Escherichia* spp. (80)		*Shigella* spp. (13), 1 taxon^‡^ (< 10 reads)	4, 0
J (837)	*Gardnerella* spp. (742)			JC polyomavirus (27), *Enhydrobacter* spp. (18)	3, 0
O (2986)		*Escherichia *spp. (1152), *Prevotella *spp.(650)	*Finegoldia* spp. (265), *Shigella* spp. (209)	*Salmonella* spp. (97), JC polyomavirus (91), *Enterobacter* spp. (87), *Klebsiella* spp. (44), *Staphylococcus *spp. (41), *Peptoniphilus* spp. (39), *Metaprevotella* spp. (30)	16, 4^¶^

Taxonomic Profiles at V2

At V2, eight samples yielded sufficient read counts for interpretation (Table 4). Among the 10 cases with read counts below the interpretable threshold at V1, six underwent SMG at V2, and all achieved read counts above the interpretable level, while the remaining taxa diversity varied across cases (Table 4 and Tables 5, 7 in Appendices). Case E exhibited a relatively balanced distribution of bacterial genera, with *Ruminococcus* spp., *Staphylococcus* spp., and *Escherichia* spp. being the most abundant. Case F exhibited only one interpretable taxon, with JCV accounting for more than 99% of all assigned reads. In Case G, *Prevotella* spp. was the most abundant, followed by *Porphyromonas* spp. Case I showed four interpretable taxa with relatively even distributions, with *Streptococcus* spp., *Prevotella* spp., and *Escherichia* spp. detected most frequently. Case J, which had the richest composition with 22 interpretable taxa, was primarily composed of *Gardnerella* spp., with JCV being the second most abundant taxon. In Case O, *Escherichia* spp. was the most abundant, followed by *Prevotella* spp.

Longitudinal Changes in Cases With Interpretable Reads at Both Visits

Of the five cases with initially interpretable read counts, three underwent follow-up SMG, but only two (Cases C and H) maintained read counts above the threshold. Consequently, reliable pre- and posttreatment microbiome comparisons were feasible in these two cases. Both cases demonstrated favorable clinical responses. In each case, the most abundant taxon remained unchanged before and after treatment, and two of the top three taxa were consistent across the time points.

Availability of data and materials

The datasets generated and/or analyzed during this study are available in the figshare repository, https://doi.org/10.6084/m9.figshare.22814834.v1.

## Discussion

Main findings

SMG was performed on 15 samples collected at V1 and nine samples collected at V2. The analysis revealed marked interindividual variability in both the HSR and taxonomic diversity. To ensure reliable taxonomic resolution, we applied a post hoc threshold of ≥10,000 HSRs; however, seven samples, particularly those with high-grade pyuria, contained fewer than 1,000 HSRs, limiting interpretability. At V1, *Lactobacillus* spp., *Gardnerella *spp., and JCV were the most frequently detected taxa. While *Lactobacillus* spp. and *Gardnerella* spp. are established constituents of the urogenital microbiome in healthy women [10-12], the frequent detection of JCV in high abundance represents a notable deviation from previous reports [18]. Conventional uropathogens such as *Escherichia *spp. and other commonly implicated bacterial genera were not identified at the interpretable level. At V2, microbial diversity remained variable among individuals, but eight samples yielded sufficient read counts for interpretation. Longitudinal comparisons between V1 and V2 were only feasible in two cases with sufficient HSR. In both cases, the predominant taxa remained unchanged after treatment.

Comparison with previous studies

To date, a metagenomic signature that definitively distinguishes causative uropathogens from commensal urinary microbiota has not yet been established. Therefore, the hasty decision to consider the most predominant taxon, such as *Lactobacillus* spp. or *Gardnerella* spp., as the causative organism is not acceptable. In prior analyses of culture-negative urine samples from patients with suspected UTIs, Hasman et al. [19] reported the detection of *Lactobacillus *spp., *Gardnerella vaginalis*, and other genera with relative abundances ranging from 15% to 53% via whole-genome sequencing. Moustafa et al. [18] used SMG and identified *Escherichia *spp., *Klebsiella *spp., *Lactobacillus *spp., and *Gardnerella *spp. as the predominant genera in some subjects, which is consistent with ACNC in this study. Li et al. [20] reported that the presence of *E.coli*, *Gardnerella* spp., and JCV were associated with pyuria. These findings are partially consistent with our results; however, the possibility that these taxa contribute to symptom development in the absence or low abundance of conventional pathogens remains unclear. Similarly, in some of our cases, *Chlamydia* spp., *Neisseria* spp., and *Ureaplasma* spp. were detected at low read counts, although their etiological significance could not be determined.

Potential role of JCV

The detection of JCV in high abundance in several cases deserves particular attention. In our study, JCV accounted for over 99% of reads in one case at V2 and exceeded 60%, even though the sample was below the interpretable level of reads at V1, suggesting possible dominance in the microbial profile. Li et al. [20] reported an association between pyuria and JCV in culture negative urine samples, and Thomas et al. reported a statistically significant association between a diagnosis of lower urinary symptoms in men and the presence of JCV [21]. Although these findings raise the possibility of JCV involvement in symptomatology, causality cannot be determined. While JCV is commonly detected in urine [22] owing to its latent infection of renal tissues, especially in immunocompromised individuals, it is not typically considered pathogenic in the lower urinary tract. Previously reported clinical manifestations of JCV infection of the bladder included hemorrhagic cystitis in immunocompromised patients [23,24]. Therefore, the relevance of JCV in acute symptoms in immunocompetent women remains unclear, and its detection in our study should be interpreted with caution.

Low Microbial Read Yield and Technical Considerations

Previous studies have reported a lower performance rate of SMG than 16S rRNA sequencing for urinary tract infection samples (approximately 50% vs. 96%) [18,25,26]. Nevertheless, SMG offers superior taxonomic resolution and a more accurate estimation of relative abundance [6-8,11,18], which justifies its utilization. However, in this study, the detection rate of interpretable microbiota using SMG was relatively low (33%). Whether this reflects the noninfectious etiologies of ACNCs or technical limitations, such as low microbial biomass or high host DNA content, remains uncertain. Notably, we observed a poor correlation between pyuria grade and SMG positivity. Samples with high leukocyte counts tended to yield a lower proportion of microbial reads, potentially because an overabundance of host DNA or microbial loads fell below the detection threshold.

Limitations

**Figure 1:**
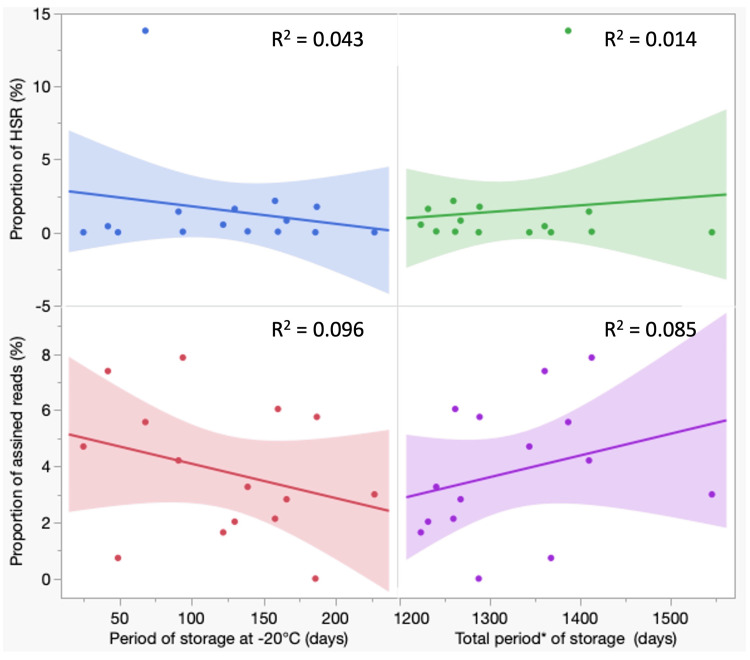
Relationship between storage duration and the proportion of HSRs or taxonomically assigned reads. HSRs: human-subtracted reads. ^*^Total period indicates sum of periods of storage at -20°C  and at -80°C.

This study had several limitations. The number of enrolled subjects was limited, and incomplete data from both visits were due to suboptimal adherence to the study protocol. The small number of paired samples with sufficient HSR at both time points limits the ability to evaluate microbiome dynamics in response to antimicrobial therapy. Midstream urine samples were used for the analysis, and there was a possibility of contamination from the genital tract. However, considering that female urinary and genital microbiomes share many common taxa, such contamination would not necessarily conflict with the objectives of this study. The culture-negative rate in this study was 7.3%, which is lower than that reported in previous studies (19-38%) [4,8,27]. This discrepancy may reflect differences in diagnostic thresholds (≥10³ CFU/mL in our study), the use of anaerobic culture methods, or the inclusion of commensal organisms as positive findings [28]. Nonetheless, given the purpose of this study to investigate the microbiome in ACNCs, bacterial loads below 10⁵ CFU/mL and nonconventional uropathogens should not be excluded from consideration. Compared with previous studies such as that of Moustafa et al. [18], the total number of sequenced reads in our samples was relatively low. However, we applied the post hoc thresholds of ≥10,000 HSRs per sample and ≥10 reads per taxon as the minimum criteria for reliable taxonomic interpretation, which are consistent with thresholds commonly used in previous studies [18,19]. At this sequencing depth, rare taxa might have been missed, although predominant taxa were likely to be detected. The shallow sequencing depth may be attributable to low microbial biomass, a high proportion of host DNA, or technical factors, such as DNA extraction efficiency and sample storage conditions. The urine samples were stored at -20 °C for 1-7 months and subsequently at -80 °C for 3-3.5 years before DNA extraction. This delay resulted from resource constraints in the testing laboratory and disruptions caused by the COVID-19 pandemic. However, no clear linear relationship was observed between storage duration and the proportion of HSRs or taxonomically assigned reads (Figure 1 in Appendices ). Therefore, although storage conditions may have contributed to some reduction of sequencing depth, comparative analysis of read counts and microbiome diversity among similarly stored samples remains interpretable within the limitations of this study.

## Conclusions

In this cohort of women presenting with ACNC, SMG revealed marked interindividual variability in microbial load and taxonomic composition. *Lactobacillus* spp., *Gardnerella* spp., and JC polyomavirus were frequently observed, whereas conventional uropathogens were not detected at interpretable levels in most cases. Reliable longitudinal comparisons were possible in only two cases; in both, the predominant taxa remained stable after treatment, suggesting that symptom resolution may not always parallel major shifts in microbiome composition. Although the limited number of interpretable samples restricts definitive conclusions, our findings support the notion that ACNC represents a heterogeneous condition that may involve unculturable abundant microbes, viral contributors, or non-microbial mechanisms. Because metagenomic signatures that can distinguish pathogens from commensals are not yet established, taxonomic predominance alone should not be assumed to indicate causality. Future studies with larger cohorts, appropriate control groups, and functional or host-response analyses are warranted to clarify the mechanisms underlying symptom development in ACNC and to improve interpretation of urinary metagenomic data.

## Appendices

**Table 5:** Metagenomic analysis of the cases at the initial and follow-up visits.

Sample_ID	Case A, V1	Case B, V1	Case C, V1	Case C, V2	Case D, V1	Case E, V1	Case E, V2	Case F, V1	Case F, V2	Case G, V1	Case G, V2	Case H, V1	Case H, V2	Case I, V1	Case I, V2	Case J, V1	Case J, V2	Case K, V1	Case L, V1	Case L, V2	Case M, V1	Case N, V1	Case O, V1	Case O, V2
all_reads	720,277	507,152	1,201,605	1584265	1,767,918	1,096,609	1,275,951	753,417	982,272	671,823	1,050,921	1,107,156	870,746	904,629	824,534	1,043,835	866,484	684,317	1,059,759	744,818	484,897	935,963	1,103,754	531,983
trimed_reads	699079	493,168	1,077,527	1,381,249	1,552,958	986,254	1,091,594	730,597	907,999	651,299	960,828	1,031,822	806,661	878,655	771,584	838,802	787,188	596,727	989,869	636,996	402,216	884,835	962,133	521,751
human_subtracted_reads	100	254	15,421	32,771	214,511	136	33,291	3,138	16,886	85	379,252	18,160	19,649	130	39,708	6829	56,197	331	21,422	340	306	14,294	5,162	115,144
hit	3	20	649	994	11,960	1	887	232	4,783	4	8,403	1,046	1,143	0	718	193	1,048	20	457	3	10	290	85	3,768
Sum of assinged reads	3	20	616	831	10,469	0	407	225	4,727	0	6,767	1,031	1,061	0	612	183	837	15	323	3	5	269	66	2,986
Species richness	3	3	31	13	17	0	57	10	6	0	45	8	12	0	37	6	22	2	24	2	3	32	19	51
Genus																								
Absiella	0	0	0	0	0	0	3	0	0	0	0	0	0	0	0	0	0	0	0	0	0	0	0	0
Acinetobacter	0	0	0	0	0	0	0	0	0	0	1	0	0	0	0	0	1	0	0	0	0	0	0	7
Actinobaculum	0	0	2	0	0	0	0	0	0	0	2	0	0	0	0	0	0	0	0	0	0	0	1	0
Actinomyces	0	0	27	1	67	0	4	0	1	0	11	0	0	0	2	0	0	0	0	0	2	19	2	0
Actinotignum	1	0	0	0	0	0	0	18	0	0	17	0	0	0	0	0	0	0	0	0	0	3	1	0
Aerococcus	0	0	1	0	2	0	0	0	0	0	2	0	0	0	0	0	0	0	9	0	0	0	1	0
Akkermansia	0	0	0	0	0	0	1	0	0	0	0	0	0	0	0	0	0	0	0	0	0	0	0	0
Alistipes	0	0	0	0	0	0	10	0	0	0	17	0	0	0	0	0	0	0	0	0	0	0	0	3
Allofustis	0	0	0	0	0	0	0	0	0	0	0	0	0	0	0	0	0	0	0	0	0	0	0	1
Alloprevotella	0	0	0	0	0	0	0	0	0	0	0	0	0	0	1	0	0	0	0	0	0	0	0	0
Alloscardovia	0	0	0	0	8	0	2	40	0	0	11	0	0	0	0	0	0	0	0	0	0	0	0	0
Anaerococcus	0	0	14	0	0	0	2	1	0	0	1	0	1	0	1	0	0	0	0	0	0	10	0	14
Anaerofustis	0	0	0	0	0	0	1	0	0	0	0	0	0	0	0	0	0	0	0	0	0	0	0	0
Anaerosphaera	0	0	20	0	0	0	0	0	0	0	0	0	0	0	0	0	0	0	0	0	0	6	1	0
Anaerostipes	0	0	0	0	0	0	1	0	0	0	0	0	0	0	0	0	0	0	0	0	0	0	0	0
Anaerotignum	0	0	0	0	0	0	1	0	0	0	0	0	0	0	0	0	0	0	0	0	0	0	0	0
Arcanobacterium	0	0	0	0	0	0	0	0	0	0	0	0	0	0	0	0	0	0	0	0	0	0	0	0
Arthrobacter	0	0	0	0	0	0	0	0	0	0	2	0	0	0	0	0	0	0	1	0	0	0	0	2
Atopobacter	0	0	0	0	0	0	0	0	0	0	0	0	0	0	0	0	0	0	0	0	0	1	0	0
Atopobium	1	0	0	0	3,252	0	0	0	0	0	7	0	0	0	0	70	0	0	0	0	0	1	2	0
Bacillus	0	0	0	0	0	0	4	0	0	0	2	0	0	0	0	0	0	0	0	0	0	0	0	9
bacterium	0	0	0	0	0	0	0	0	0	0	0	0	0	0	0	0	0	0	0	0	0	0	0	0
Bacteroidales	0	0	0	0	0	0	0	0	0	0	0	0	0	0	0	0	0	0	0	0	0	0	0	0
Bacteroides	0	0	7	0	0	0	41	0	0	0	40	0	0	0	1	0	0	0	1	0	0	8	1	5
Bacteroidetes	0	0	0	0	0	0	0	0	0	0	0	0	0	0	0	0	0	0	0	0	0	0	0	0
Barnesiella	0	0	0	1	0	0	1	0	0	0	2	0	0	0	0	0	0	0	0	0	0	0	0	0
Bifidobacteriaceae	0	0	0	0	0	0	0	0	0	0	0	0	0	0	0	0	0	0	0	0	0	0	0	0
Bifidobacterium	0	0	0	0	0	0	0	0	0	0	0	0	0	0	0	0	0	0	0	0	0	0	0	0
Blastomonas	0	0	0	0	0	0	0	0	0	0	0	0	0	0	1	0	3	0	0	0	0	0	0	0
Blautia	0	0	0	0	0	0	2	0	0	0	6	0	0	0	0	0	0	0	0	0	0	0	0	0
Brevibacterium	0	0	0	0	0	0	0	0	0	0	0	0	0	0	0	0	0	0	0	0	0	0	0	0
Brevundimonas	0	0	0	0	0	0	0	0	0	0	0	0	0	0	0	0	1	0	0	0	0	0	0	0
Bulleidia	0	0	0	0	0	0	0	0	0	0	0	0	0	0	1	0	0	0	0	0	0	1	0	0
Butyricicoccus	0	0	0	0	0	0	0	0	0	0	1	0	0	0	0	0	0	0	0	0	0	0	0	0
Campylobacter	0	0	4	0	0	0	0	0	0	0	1	0	0	0	3	0	0	0	0	0	0	1	0	0
Canis	0	0	0	0	0	0	0	0	0	0	0	0	0	0	0	0	0	0	0	0	0	0	0	0
Chlamydia	0	0	0	0	0	0	0	0	0	0	0	12	11	0	0	0	0	0	6	0	0	0	0	0
Citrobacter	0	0	0	0	0	0	9	0	0	0	0	0	0	0	0	0	0	0	0	0	0	0	0	0
Clostridia	0	0	0	0	0	0	0	0	0	0	0	0	0	0	0	0	0	0	0	0	0	0	0	0
Clostridiaceae	0	0	0	0	0	0	0	0	0	0	0	0	0	0	0	0	0	0	0	0	0	0	0	0
Clostridiales	0	0	0	0	0	0	0	0	0	0	0	0	0	0	0	0	0	0	0	0	0	0	0	0
Clostridioides	0	0	0	0	0	0	0	0	0	0	0	0	0	0	0	1	0	0	0	0	0	0	0	0
Clostridium	0	0	0	0	0	0	9	0	0	0	0	0	0	0	0	0	0	0	0	0	0	0	0	0
Collinsella	0	0	1	0	0	0	0	0	0	0	16	0	0	0	0	0	0	0	0	0	0	0	0	0
Coprobacillus	0	0	0	0	0	0	0	0	0	0	0	0	0	0	0	0	0	0	0	0	0	0	0	0
Coprobacter	0	0	0	0	0	0	0	0	0	0	1	0	0	0	0	0	0	0	0	0	0	0	0	0
Coriobacteriaceae	0	0	0	0	0	0	0	0	0	0	0	0	0	0	0	0	0	0	0	0	0	0	0	0
Coriobacteriales	0	0	0	0	0	0	0	0	0	0	0	0	0	0	0	0	0	0	0	0	0	0	0	0
Corynebacterium	0	0	0	0	0	0	0	0	0	0	0	0	0	0	0	0	0	0	0	0	0	0	0	0
Criibacterium	0	0	0	0	0	0	0	0	0	0	0	0	0	0	0	0	0	0	0	0	0	0	0	0
Croceicoccus	0	0	0	0	0	0	0	0	0	0	0	0	0	0	0	0	1	0	0	0	0	0	0	0
Cutibacterium	0	0	0	0	0	0	0	0	0	0	0	0	0	0	0	0	0	0	0	0	0	0	0	0
Deinococcus	0	0	0	0	0	0	0	0	0	0	0	0	0	0	0	0	2	0	0	0	0	0	0	0
Dermabacter	0	0	0	0	0	0	0	0	0	0	0	0	0	0	0	0	0	0	0	0	0	0	0	0
Dermacoccus	0	0	0	0	0	0	0	0	0	0	0	0	0	0	0	0	0	0	0	0	0	0	0	2
Dialister	0	0	1	0	13	0	1	0	0	0	0	1	0	0	0	0	0	0	0	0	0	3	1	0
Dorea	0	0	0	0	0	0	1	0	0	0	0	0	0	0	0	0	0	0	0	0	0	0	0	0
Drancourtella	0	0	0	0	0	0	2	0	0	0	0	0	0	0	0	0	0	0	0	0	0	0	0	0
Eisenbergiella	0	0	0	0	0	0	1	0	0	0	0	0	0	0	0	0	0	0	0	0	0	0	0	0
Enhydrobacter	0	0	0	0	0	0	0	0	0	0	0	0	0	0	0	0	18	0	1	0	0	0	0	1
Enorma	0	0	0	0	0	0	0	0	0	0	0	0	0	0	0	0	0	0	0	0	0	0	0	1
Enterobacter	0	0	0	0	0	0	3	0	0	0	0	0	0	0	6	0	0	0	0	0	0	1	0	87
Enterobacteria	0	0	0	0	0	0	3	0	0	0	0	0	0	0	4	0	0	0	0	0	0	0	0	87
Enterococcus	0	0	0	0	0	0	42	1	0	0	0	0	6	0	0	0	0	0	0	0	0	0	0	8
Erwinia	0	0	0	0	0	0	0	0	0	0	0	0	0	0	0	0	0	0	0	0	0	0	0	3
Erysipelatoclostridium	0	0	0	0	0	0	3	0	0	0	0	0	0	0	0	0	0	0	0	0	0	0	0	0
Erythrobacteraceae	0	0	0	0	0	0	0	0	0	0	0	0	0	0	0	0	0	0	0	0	0	0	0	0
Escherichia	0	0	2	0	0	0	47	0	0	0	1	0	0	0	80	0	0	0	1	0	0	0	0	1,152
Eubacterium	0	0	0	0	0	0	0	0	0	0	0	0	0	0	0	0	0	0	0	0	0	0	0	0
Exiguobacterium	0	0	0	0	0	0	0	0	0	0	0	0	0	0	0	0	0	0	0	0	0	0	0	0
Ezakiella	0	0	0	0	0	0	0	0	0	0	0	0	0	0	0	0	2	0	0	0	0	16	0	3
Facklamia	0	0	0	0	0	0	0	0	0	0	1	0	0	0	4	0	0	0	1	0	0	8	0	6
Faecalibacterium	0	0	0	0	0	0	0	0	0	0	0	0	0	0	0	0	0	0	0	0	0	0	0	0
Faecalitalea	0	0	0	0	0	0	1	0	0	0	1	0	0	0	0	0	0	0	1	0	0	0	0	0
Fastidiosipila	0	0	1	0	0	0	0	0	0	0	0	0	0	0	0	0	0	0	0	0	0	1	0	0
Fenollaria	0	0	4	0	0	0	0	0	0	0	0	0	0	0	0	0	0	0	0	0	0	1	0	11
Filifactor	0	0	0	0	0	0	0	0	0	0	0	0	0	0	0	0	0	0	0	0	0	0	0	0
Finegoldia	0	0	9	0	0	0	24	0	1	0	0	0	0	0	2	0	1	0	1	0	0	0	1	265
Firmicutes	0	0	0	0	0	0	0	0	0	0	0	0	0	0	0	0	0	0	0	0	0	0	0	0
Flavonifractor	0	0	0	0	0	0	1	0	0	0	0	0	0	0	0	0	0	0	0	0	0	0	0	0
Fusobacterium	0	0	0	0	0	0	0	0	0	0	0	0	0	0	0	0	0	0	0	0	0	0	0	0
Gardnerella	1	0	0	0	6,978	0	0	1	0	0	0	0	1	0	0	82	742	12	35	0	1	1	0	6
Gemella	0	0	2	0	0	0	1	0	0	0	0	0	0	0	2	0	0	0	0	0	0	0	0	0
Gordonibacter	0	0	0	0	0	0	0	0	0	0	0	0	0	0	0	0	0	0	0	0	0	0	0	0
Halomonas	0	0	0	0	0	0	0	0	0	0	0	0	0	0	0	0	1	0	0	0	0	0	0	1
Helcococcus	0	0	0	0	0	0	0	0	0	0	0	0	0	0	0	0	0	0	0	0	0	0	0	0
Human	0	0	0	0	0	0	0	0	0	0	0	0	0	0	0	0	0	0	0	0	0	0	0	0
Ileibacterium	0	0	0	0	0	0	0	0	0	0	0	0	0	0	0	0	0	0	0	0	0	0	0	0
Janibacter	0	0	0	0	0	0	0	0	0	0	0	0	0	0	0	0	0	0	0	0	0	0	0	1
Janthinobacterium	0	0	0	0	0	0	0	0	0	0	0	0	0	0	0	0	0	0	0	0	0	0	0	0
JC polyomavirus	0	16	318	456	1	0	0	135	4,717	0	13	0	0	0	1	12	27	0	21	1	2	0	3	91
Jonquetella	0	0	0	0	0	0	0	0	0	0	0	0	0	0	0	0	0	0	0	0	0	1	1	0
Kallipyga	0	0	0	0	0	0	0	0	0	0	0	0	0	0	0	0	0	0	0	0	0	0	0	0
Khoudiadiopia	0	0	0	0	1	0	0	0	0	0	0	0	0	0	0	0	0	0	0	0	0	1	0	4
Klebsiella	0	0	0	0	0	0	1	0	0	0	0	0	0	0	5	0	0	0	0	0	0	0	0	44
Kocuria	0	0	0	0	0	0	0	0	0	0	1	0	0	0	1	0	0	0	0	0	0	0	0	0
Lachnoclostridium	0	0	0	0	0	0	1	0	0	0	0	0	0	0	0	0	0	0	0	0	0	0	0	0
Lachnospiraceae	0	0	0	0	0	0	0	0	0	0	0	0	0	0	0	0	0	0	0	0	0	0	0	0
Lactobacillus	0	3	0	0	16	0	2	0	0	0	6	999	993	0	0	17	0	3	225	2	0	2	0	7
Lactococcus	0	0	0	0	0	0	0	0	0	0	0	0	0	0	0	0	0	0	0	0	0	0	0	3
Lagierella	0	0	0	0	0	0	0	0	0	0	0	0	0	0	0	0	0	0	0	0	0	0	0	0
Lawsonella	0	0	1	1	0	0	0	0	0	0	29	0	0	0	1	0	0	0	0	0	0	0	0	0
Legionella	0	0	0	0	0	0	0	0	0	0	0	0	0	0	0	0	0	0	0	0	0	0	0	2
Leptotrichia	0	0	0	0	0	0	0	0	0	0	0	0	0	0	0	0	0	0	0	0	0	0	0	0
Leucobacter	0	0	0	0	0	0	0	0	0	0	0	0	0	0	0	0	0	0	0	0	0	0	0	3
Leuconostoc	0	0	0	0	0	0	0	0	0	0	0	0	0	0	0	0	0	0	0	0	0	0	0	3
Levyella	0	0	1	1	0	0	1	0	1	0	0	0	0	0	2	0	0	0	0	0	0	0	0	0
Libanicoccus	0	0	0	0	0	0	0	0	0	0	2	0	0	0	0	0	0	0	0	0	0	0	0	0
Lysobacter	0	0	0	0	0	0	0	0	0	0	0	0	0	0	1	0	0	0	0	0	0	0	0	0
Mageeibacillus	0	0	0	0	5	0	0	0	0	0	0	0	0	0	0	0	0	0	0	0	0	0	0	0
Malassezia	0	0	0	0	0	0	0	0	0	0	0	0	0	0	0	0	0	0	1	0	0	0	0	0
Megasphaera	0	0	1	0	0	0	1	0	0	0	0	0	0	0	0	0	0	0	0	0	0	0	0	0
Merdibacter	0	0	0	0	0	0	1	0	0	0	0	0	0	0	0	0	0	0	0	0	0	0	0	0
Merdimonas	0	0	0	0	0	0	1	0	0	0	0	0	0	0	0	0	0	0	0	0	0	0	0	0
Merkel cell polyomavirus	0	0	0	6	0	0	0	0	0	0	0	0	0	0	0	0	0	0	0	0	0	0	0	0
Metaprevotella	0	0	23	71	6	0	0	1	0	0	508	0	2	0	7	0	0	0	0	0	0	1	1	30
Methylobacillus	0	0	0	0	0	0	1	0	0	0	0	0	0	0	0	0	0	0	0	0	0	0	0	4
Methylobacterium	0	0	0	0	0	0	0	0	0	0	0	0	0	0	0	0	0	0	0	0	0	0	0	0
Methylophilus	0	0	0	0	0	0	0	0	0	0	0	0	0	0	2	0	6	0	2	0	0	0	0	0
Methylorubrum	0	0	0	0	0	0	0	0	0	0	0	0	1	0	3	0	5	0	1	0	0	0	0	0
Methylovorus	0	0	0	0	0	0	0	0	0	0	0	0	0	0	0	0	0	0	0	0	0	0	0	2
Micrococcus	0	0	0	0	0	0	0	0	0	0	0	0	1	0	0	0	0	0	0	0	0	0	0	0
Mobiluncus	0	0	2	0	0	0	0	0	0	0	3	0	0	0	0	0	0	0	0	0	0	0	1	0
Mogibacterium	0	0	0	0	0	0	0	0	0	0	0	0	0	0	0	0	0	0	0	0	0	0	0	0
Moraxella	0	0	0	0	0	0	0	0	0	0	0	0	0	0	0	0	8	0	0	0	0	0	0	0
Murdochiella	0	0	0	0	0	0	0	0	0	0	0	0	0	0	0	0	0	0	0	0	0	1	0	0
Muribaculaceae	0	0	0	0	0	0	0	0	0	0	0	0	0	0	0	0	0	0	0	0	0	0	0	0
Mycobacteroides	0	0	0	0	0	0	1	0	0	0	0	0	0	0	0	0	0	0	0	0	0	0	0	0
Mycoplasma	0	0	0	0	0	0	0	0	0	0	0	1	0	0	0	0	0	0	0	0	0	0	0	4
Ndongobacter	0	0	0	0	0	0	0	0	0	0	0	0	0	0	0	0	0	0	0	0	0	0	0	0
Negativicoccus	0	0	0	0	0	0	1	0	0	0	0	0	0	0	0	0	0	0	0	0	0	0	0	0
Neisseria	0	0	1	0	0	0	1	0	0	0	2	10	0	0	3	0	1	0	2	0	0	0	0	7
Neofamilia	0	0	0	0	0	0	0	0	0	0	0	0	0	0	0	0	0	0	0	0	0	0	0	0
Novosphingobium	0	0	0	0	0	0	0	0	0	0	0	0	0	0	0	0	0	0	1	0	0	0	0	0
Olegusella	0	0	0	0	0	0	0	0	0	0	0	0	0	0	0	0	0	0	0	0	0	0	0	0
Olsenella	0	0	0	0	0	0	0	0	0	0	1	0	0	0	0	0	0	0	0	0	0	0	0	0
Ornithobacterium	0	0	0	0	0	0	0	0	0	0	0	0	0	0	0	0	0	0	0	0	0	0	0	0
Oscillibacter	0	0	0	0	0	0	1	0	0	0	1	0	0	0	0	0	0	0	0	0	0	0	0	0
Oscillospiraceae	0	0	0	0	0	0	0	0	0	0	0	0	0	0	0	0	0	0	0	0	0	0	0	0
Pan	0	0	0	0	0	0	0	0	0	0	0	0	0	0	0	0	0	0	0	0	0	0	0	0
Parabacteroides	0	0	0	0	0	0	4	0	0	0	0	0	0	0	0	0	0	0	0	0	0	0	0	0
Paracoccus	0	0	0	0	0	0	0	0	0	0	0	0	0	0	0	0	0	0	0	0	0	0	0	0
Parolsenella	0	0	0	2	0	0	0	0	0	0	4	0	0	0	0	0	0	0	0	0	0	0	0	0
Parvimonas	0	0	0	0	0	0	0	0	0	0	0	0	0	0	0	0	0	0	0	0	0	1	0	0
Peptococcus	0	0	0	0	0	0	0	0	0	0	0	0	0	0	0	0	0	0	0	0	0	1	0	0
Peptoniphilus	0	0	14	0	7	0	9	0	2	0	0	0	0	0	2	0	1	0	0	0	0	30	0	39
Peptostreptococcaceae	0	0	0	0	0	0	0	0	0	0	0	0	0	0	0	0	0	0	0	0	0	0	0	0
Peptostreptococcus	0	0	0	0	0	0	0	0	0	0	0	0	0	0	0	0	0	0	0	0	0	1	0	0
Phoenicibacter	0	0	0	1	0	0	0	0	0	0	4	0	0	0	0	0	0	0	0	0	0	0	0	0
Phreatobacter	0	0	0	0	0	0	0	0	0	0	0	0	0	0	1	0	4	0	2	0	0	0	0	0
Piscirickettsia	0	0	0	0	0	0	0	0	0	0	0	0	0	0	0	0	0	0	0	0	0	0	0	7
Plasmid	0	0	0	0	0	0	0	0	0	0	0	0	0	0	0	0	0	0	0	0	0	0	0	0
Porphyrobacter	0	0	0	0	0	0	0	0	0	0	0	0	0	0	0	0	1	0	0	0	0	0	0	0
Porphyromonadaceae	0	0	0	0	0	0	0	0	0	0	0	0	0	0	0	0	0	0	0	0	0	0	0	0
Porphyromonas	0	0	38	42	2	0	4	0	0	0	2,446	0	0	0	1	0	0	0	0	0	0	105	20	6
Prevotella	0	0	95	238	99	0	4	22	0	0	2,869	4	4	0	126	0	0	0	1	0	0	5	17	650
Prevotellamassilia	0	0	0	0	0	0	1	0	0	0	0	0	0	0	0	0	0	0	0	0	0	0	0	0
Propionibacterium	0	0	0	0	0	0	0	0	0	0	0	0	0	0	0	0	0	0	0	0	0	0	0	0
Propionimicrobium	0	1	9	9	0	0	1	5	0	0	671	0	0	0	0	0	0	0	1	0	0	26	5	0
Proteus	0	0	0	0	0	0	0	0	0	0	0	0	0	0	0	0	1	0	0	0	0	0	0	0
Pseudoglutamicibacter	0	0	0	0	0	0	1	0	0	0	3	0	0	0	0	0	0	0	0	0	0	0	0	0
Pseudomonas	0	0	1	0	0	0	0	0	0	0	0	0	0	0	2	0	0	0	0	0	0	0	0	0
Psychrobacter	0	0	0	0	0	0	0	0	0	0	0	0	0	0	0	0	1	0	0	0	0	0	0	0
Rhodococcus	0	0	0	0	0	0	0	0	0	0	0	0	0	0	0	0	0	0	0	0	0	0	0	4
Roseburia	0	0	0	0	0	0	0	0	0	0	1	0	0	0	0	0	0	0	0	0	0	0	0	0
Rothia	0	0	0	0	0	0	0	0	0	0	2	0	0	0	1	0	0	0	0	0	0	0	0	0
Ruminococcaceae	0	0	0	0	0	0	0	0	0	0	0	0	0	0	0	0	0	0	0	0	0	0	0	0
Ruminococcus	0	0	0	0	0	0	64	0	0	0	10	0	0	0	0	0	0	0	0	0	0	0	0	7
Salmonella	0	0	0	0	0	0	7	0	0	0	0	0	0	0	1	0	0	0	0	0	0	0	0	97
Shigella	0	0	0	0	0	0	3	0	0	0	0	0	0	0	13	0	0	0	1	0	0	0	0	209
Slackia	0	0	0	0	0	0	0	0	0	0	0	0	0	0	0	0	0	0	0	0	0	0	0	0
Sneathia	0	0	0	0	0	0	0	0	0	0	0	0	0	0	0	0	0	0	0	0	0	0	0	0
Sphingobium	0	0	0	0	0	0	0	0	0	0	0	0	0	0	0	0	0	0	1	0	0	0	0	0
Sphingomonas	0	0	0	0	0	0	0	0	0	0	0	0	0	0	5	0	5	0	5	0	0	0	0	0
Staphylococcus	0	0	1	0	1	0	48	0	0	0	0	0	10	0	2	1	5	0	0	0	0	0	0	41
Streptobacillus	0	0	0	0	0	0	1	0	0	0	0	0	0	0	0	0	0	0	0	0	0	0	0	17
Streptococcus	0	0	4	2	1	0	19	1	0	0	27	0	1	0	318	0	0	0	0	0	0	1	2	6
Stx2-converting	0	0	0	0	0	0	0	0	0	0	0	0	0	0	0	0	0	0	0	0	0	0	0	0
Subdoligranulum	0	0	0	0	0	0	2	0	0	0	0	0	0	0	0	0	0	0	0	0	0	0	0	0
Tannerella	0	0	0	0	0	0	0	0	0	0	1	0	0	0	0	0	0	0	0	0	0	0	0	0
Tissierellia	0	0	0	0	0	0	0	0	0	0	0	0	0	0	0	0	0	0	0	0	0	0	0	0
Treponema	0	0	0	0	0	0	0	0	0	0	0	0	0	0	0	0	0	0	0	0	0	0	1	0
Trueperella	0	0	0	0	0	0	0	0	0	0	2	1	0	0	0	0	0	0	0	0	0	0	0	0
Tyzzerella	0	0	0	0	0	0	1	0	0	0	0	0	0	0	0	0	0	0	0	0	0	0	0	0
uncultured	0	0	0	0	0	0	2	0	0	0	0	0	0	0	0	0	0	0	0	0	0	0	0	0
Ureaplasma	0	0	2	0	0	0	0	0	0	0	0	3	30	0	4	0	0	0	2	0	0	0	0	4
Urinacoccus	0	0	0	0	0	0	0	0	0	0	0	0	0	0	0	0	0	0	0	0	0	3	0	1
Urmitella	0	0	2	0	0	0	0	0	0	0	0	0	0	0	0	0	0	0	0	0	0	1	0	0
Varibaculum	0	0	8	0	0	0	0	0	0	0	17	0	0	0	0	0	0	0	0	0	0	8	4	1
Veillonella	0	0	0	0	10	0	0	0	5	0	0	0	0	0	1	0	0	0	0	0	0	0	0	12
Veillonellaceae	0	0	0	0	0	0	0	0	0	0	0	0	0	0	0	0	0	0	0	0	0	0	0	0
Vibrio	0	0	0	0	0	0	0	0	0	0	0	0	0	0	1	0	0	0	0	0	0	0	0	0
Weeksella	0	0	0	0	0	0	0	0	0	0	1	0	0	0	0	0	0	0	0	0	0	0	0	0
Yersinia	0	0	0	0	0	0	2	0	0	0	0	0	0	0	0	0	0	0	0	0	0	0	0	6
Not assinged	0	0	33	163	1,491	1	480	7	56	4	1,636	15	82	0	106	10	211	5	134	0	5	21	19	782

**Table 6:** Abundance-based rank of taxa idenfified in each case at the initial visit. Cases E, G, and I have no assigned reads.

Case A			Case B			Case C			Case D			Case F			Case H			Case J			Case K			Case L			Case M			Case N			Case O		
	Assigned reads	Relative abundance		Assigned reads	Relative abundance		Assigned reads	Relative abundance		Assigned reads	Relative abundance		Assigned reads	Relative abundance		Assigned reads	Relative abundance		Assigned reads	Relative abundance		Assigned reads	Relative abundance		Assigned reads	Relative abundance		Assigned reads	Relative abundance		Assigned reads	Relative abundance		Assigned reads	Relative abundance
Gardnerella	1	33.3	JC polyomavirus	16	80	JC polyomavirus	318	51.6	Gardnerella	6,978	66.7	JC polyomavirus	135	60	Lactobacillus	999	96.9	Gardnerella	82	44.8	Gardnerella	12	80	Lactobacillus	225	69.9	JC polyomavirus	2	40	Porphyromonas	105	39	Porphyromonas	20	30.3
Atopobium	1	33.3	Lactobacillus	3	15	Prevotella	95	15.4	Atopobium	3,252	31.1	Alloscardovia	40	17.8	Chlamydia	12	1.2	Atopobium	70	38.3	Lactobacillus	3	20	Gardnerella	35	10.9	Actinomyces	2	40	Peptoniphilus	30	11.2	Prevotella	17	25.8
			Propionimicrobium	1	5	Porphyromonas	38	6.2	Prevotella	99	0.9	Prevotella	22	9.8	Neisseria	10	1	Lactobacillus	17	9.3				JC polyomavirus	21	6.5	Gardnerella	1	20	Propionimicrobium	26	9.7	Propionimicrobium	5	7.6
						Actinomyces	27	4.4	Actinomyces	67	0.6	Actinotignum	18	8	Prevotella	4	0.4	JC polyomavirus	12	6.6				Aerococcus	9	2.8				Actinomyces	19	7.1	Varibaculum	4	6.1
						Metaprevotella	23	3.7	Lactobacillus	16	0.2	Propionimicrobium	5	2.2	Ureaplasma	3	0.3	Staphylococcus	1	0.5				Chlamydia	6	1.9				Ezakiella	16	5.9	JC polyomavirus	3	4.5
						Anaerosphaera	20	3.2	Dialister	13	0.1	Gardnerella	1	0.4	Dialister	1	0.1	Clostridioides	1	0.5				Sphingomonas	5	1.6				Anaerococcus	10	3.7	Actinomyces	2	3
						Peptoniphilus	14	2.3	Veillonella	10	0.1	Metaprevotella	1	0.4	Mycoplasma	1	0.1							Neisseria	2	0.6				Bacteroides	8	3	Atopobium	2	3
						Anaerococcus	14	2.3	Alloscardovia	8	0.1	Streptococcus	1	0.4	Trueperella	1	0.1							Ureaplasma	2	0.6				Facklamia	8	3	Streptococcus	2	3
						Propionimicrobium	9	1.5	Peptoniphilus	7	0.1	Anaerococcus	1	0.4										Methylophilus	2	0.6				Varibaculum	8	3	Bacteroides	1	1.5
						Finegoldia	9	1.5	Metaprevotella	6	0.1	Enterococcus	1	0.4										Phreatobacter	2	0.6				Anaerosphaera	6	2.2	Anaerosphaera	1	1.5
						Varibaculum	8	1.3	Mageeibacillus	5	0													Prevotella	1	0.3				Prevotella	5	1.9	Dialister	1	1.5
						Bacteroides	7	1.1	Porphyromonas	2	0													Propionimicrobium	1	0.3				Dialister	3	1.1	Actinotignum	1	1.5
						Streptococcus	4	0.6	Aerococcus	2	0													Finegoldia	1	0.3				Actinotignum	3	1.1	Metaprevotella	1	1.5
						Campylobacter	4	0.6	JC polyomavirus	1	0													Bacteroides	1	0.3				Urinacoccus	3	1.1	Jonquetella	1	1.5
						Fenollaria	4	0.6	Streptococcus	1	0													Escherichia	1	0.3				Lactobacillus	2	0.7	Aerococcus	1	1.5
						Actinobaculum	2	0.3	Staphylococcus	1	0													Arthrobacter	1	0.3				Gardnerella	1	0.4	Finegoldia	1	1.5
						Escherichia	2	0.3	Khoudiadiopia	1	0													Enhydrobacter	1	0.3				Atopobium	1	0.4	Actinobaculum	1	1.5
						Gemella	2	0.3																Facklamia	1	0.3				Metaprevotella	1	0.4	Mobiluncus	1	1.5
						Mobiluncus	2	0.3																Faecalitalea	1	0.3				Streptococcus	1	0.4	Treponema	1	1.5
						Ureaplasma	2	0.3																Malassezia	1	0.3				Khoudiadiopia	1	0.4			
						Urmitella	2	0.3																Methylorubrum	1	0.3				Campylobacter	1	0.4			
						Dialister	1	0.2																Shigella	1	0.3				Fenollaria	1	0.4			
						Aerococcus	1	0.2																Sphingobium	1	0.3				Urmitella	1	0.4			
						Staphylococcus	1	0.2																						Fastidiosipila	1	0.4			
						Collinsella	1	0.2																						Atopobacter	1	0.4			
						Fastidiosipila	1	0.2																						Bulleidia	1	0.4			
						Lawsonella	1	0.2																						Enterobacter	1	0.4			
						Levyella	1	0.2																						Jonquetella	1	0.4			
						Megasphaera	1	0.2																						Murdochiella	1	0.4			
						Neisseria	1	0.2																						Parvimonas	1	0.4			
						Pseudomonas	1	0.2																						Peptococcus	1	0.4			
																														Peptostreptococcus	1	0.4			

**Table 7:** Abundance-based rank of taxa idenfified in each case at the follow-up visit.

Case C			Case E			Case F			Case G			Case H			Case I			Case J			Case L			Case O		
	Assigned reads	Relative abundance		Assigned reads	Relative abundance		Assigned reads	Relative abundance		Assigned reads	Relative abundance		Assigned reads	Relative abundance		Assigned reads	Relative abundance		Assigned reads	Relative abundance		Assigned reads	Relative abundance		Assigned reads	Relative abundance
JC polyomavirus	456		Ruminococcus	64		JC polyomavirus	4717		Prevotella	2869		Lactobacillus	993		Streptococcus	318		Gardnerella	742		Lactobacillus	2		Escherichia	1152	
Prevotella	238		Staphylococcus	48		Veillonella	5		Porphyromonas	2446		Ureaplasma	30		Prevotella	126		JC polyomavirus	27		JC polyomavirus	1		Prevotella	650	
Metaprevotella	71		Escherichia	47		Peptoniphilus	2		Propionimicrobium	671		Chlamydia	11		Escherichia	80		Enhydrobacter	18					Finegoldia	265	
Porphyromonas	42		Enterococcus	42		Actinomyces	1		Metaprevotella	508		Staphylococcus	10		Shigella	13		Moraxella	8					Shigella	209	
Propionimicrobium	9		Bacteroides	41		Finegoldia	1		Bacteroides	40		Enterococcus	6		Metaprevotella	7		Methylophilus	6					Salmonella	97	
Merkel cell polyomavirus	6		Finegoldia	24		Levyella	1		Lawsonella	29		Prevotella	4		Enterobacter	6		Methylorubrum	5					JC polyomavirus	91	
Parolsenella	2		Streptococcus	19					Streptococcus	27		Metaprevotella	2		Klebsiella	5		Sphingomonas	5					Enterobacter	87	
Streptococcus	2		Alistipes	10					Actinotignum	17		Anaerococcus	1		Sphingomonas	5		Staphylococcus	5					Enterobacteria	87	
Actinomyces	1		Citrobacter	9					Alistipes	17		Gardnerella	1		Enterobacteria	4		Phreatobacter	4					Klebsiella	44	
Barnesiella	1		Clostridium	9					Varibaculum	17		Methylorubrum	1		Facklamia	4		Blastomonas	3					Staphylococcus	41	
Lawsonella	1		Peptoniphilus	9					Collinsella	16		Micrococcus	1		Ureaplasma	4		Deinococcus	2					Peptoniphilus	39	
Levyella	1		Salmonella	7					JC polyomavirus	13		Streptococcus	1		Campylobacter	3		Ezakiella	2					Metaprevotella	30	
Phoenicibacter	1		Actinomyces	4					Actinomyces	11					Methylorubrum	3		Acinetobacter	1					Streptobacillus	17	
			Bacillus	4					Alloscardovia	11					Neisseria	3		Brevundimonas	1					Anaerococcus	14	
			Parabacteroides	4					Ruminococcus	10					Actinomyces	2		Croceicoccus	1					Veillonella	12	
			Porphyromonas	4					Atopobium	7					Finegoldia	2		Finegoldia	1					Fenollaria	11	
			Prevotella	4					Blautia	6					Gemella	2		Halomonas	1					Bacillus	9	
			Absiella	3					Lactobacillus	6					Levyella	2		Neisseria	1					Enterococcus	8	
			Enterobacter	3					Parolsenella	4					Methylophilus	2		Peptoniphilus	1					Acinetobacter	7	
			Enterobacteria	3					Phoenicibacter	4					Peptoniphilus	2		Porphyrobacter	1					Lactobacillus	7	
			Erysipelatoclostridium	3					Mobiluncus	3					Pseudomonas	2		Proteus	1					Neisseria	7	
			Shigella	3					Pseudoglutamicibacter	3					Staphylococcus	2		Psychrobacter	1					Piscirickettsia	7	
			Alloscardovia	2					Actinobaculum	2					Alloprevotella	1								Ruminococcus	7	
			Anaerococcus	2					Aerococcus	2					Anaerococcus	1								Facklamia	6	
			Blautia	2					Arthrobacter	2					Bacteroides	1								Gardnerella	6	
			Drancourtella	2					Bacillus	2					Blastomonas	1								Porphyromonas	6	
			Lactobacillus	2					Barnesiella	2					Bulleidia	1								Streptococcus	6	
			Subdoligranulum	2					Libanicoccus	2					JC polyomavirus	1								Yersinia	6	
			Yersinia	2					Neisseria	2					Kocuria	1								Bacteroides	5	
			Akkermansia	1					Rothia	2					Lawsonella	1								Khoudiadiopia	4	
			Anaerofustis	1					Trueperella	2					Lysobacter	1								Methylobacillus	4	
			Anaerostipes	1					Acinetobacter	1					Phreatobacter	1								Mycoplasma	4	
			Anaerotignum	1					Anaerococcus	1					Porphyromonas	1								Rhodococcus	4	
			Barnesiella	1					Butyricicoccus	1					Rothia	1								Ureaplasma	4	
			Dialister	1					Campylobacter	1					Salmonella	1								Alistipes	3	
			Dorea	1					Coprobacter	1					Veillonella	1								Erwinia	3	
			Eisenbergiella	1					Escherichia	1					Vibrio	1								Ezakiella	3	
			Faecalitalea	1					Facklamia	1														Lactococcus	3	
			Flavonifractor	1					Faecalitalea	1														Leucobacter	3	
			Gemella	1					Kocuria	1														Leuconostoc	3	
			Klebsiella	1					Olsenella	1														Arthrobacter	2	
			Lachnoclostridium	1					Oscillibacter	1														Dermacoccus	2	
			Levyella	1					Roseburia	1														Legionella	2	
			Megasphaera	1					Tannerella	1														Methylovorus	2	
			Merdibacter	1					Weeksella	1														Allofustis	1	
			Merdimonas	1																				Enhydrobacter	1	
			Methylobacillus	1																				Enorma	1	
			Mycobacteroides	1																				Halomonas	1	
			Negativicoccus	1																				Janibacter	1	
			Neisseria	1																				Urinacoccus	1	
			Oscillibacter	1																				Varibaculum	1	
			Prevotellamassilia	1																						
			Propionimicrobium	1																						
			Pseudoglutamicibacter	1																						
			Streptobacillus	1																						
			Tyzzerella	1																						
